# Supplementary material for: Orthorexia nervosa and social media: A mixed-methods scoping review using a systematic methodology
Source: PLoS One. 2026 Mar 11;21(3):e0340219. doi: 10.1371/journal.pone.0340219 (PMC12978498; doi:10.1371/journal.pone.0340219)
Supplement: Attachment — Submitted filename: Response to Reviewers.docx [file pone.0340219.s004.docx]

**Thank you for taking the time to review our manuscript. We greatly appreciate your efforts and consideration. We have adjusted the manuscript to accommodate each recommendation of the reviewer and believe this has led to considerably improvement. Therefore, we want to thank the reviewer for their valuable advice. In the remainder of this letter, we will explain and describe how we have addressed each comment. (The answer to each comment, addressed, can be found below.)**

Reviewer #1: Dear Authors,
The manuscript presents a comprehensive scoping review on the relationship between orthorexia nervosa (ON) and social media. The topic is highly relevant and addresses a growing gap in the health sciences literature. The authors structured the study according to PRISMA-ScR guidelines, registered their protocol on OSF, and performed a wide database search, which demonstrates methodological transparency.

Recommendations

1. The majority of included studies are cross-sectional, limiting causal inference. While this is noted, the discussion should provide clearer recommendations for future longitudinal and experimental designs.

**Thank you for this valuable suggestion. Specific recommendations were added as suggested (see Page 19 lines 397 to 408):**
*“The included studies were observational and cross-sectional, indicating the scarcity of experimental studies and limiting the ability to synthesize findings identifying a cause-effect relationship between ON and social media. As previously presented, social media platforms can potentially have a mitigating or distressing effect on ON.(40) Experimental studies can help pinpoint the role of potential moderators such as social media use duration, content and platforms on ON symptomatology. Also, experimental designs where exposure to healthy food-related content on social media, as opposed to other unrelated content, can help determine potential causality. Adding to that, longitudinal studies can be conducted to assess the temporal frame of the relationship between ON and social media, tracking the progression or changes in orthorexic tendencies in parallel with social media use. Also, it can help identify different trajectories and profiles of participants and their characteristics through longitudinal designs.”*

2. The limitations of measurement tools (e.g., ORTO-11, ORTO-15) regarding validity and reliability should be emphasized more strongly.

**The limitations of the measurement tools were elaborated** (see page 24 lines 514 to 521**:**
*“**The scales used in the included studies differed, while most used the ORTO-11 and ORTO-15. However, the cut-off point of these two scales was inconsistent across studies.^22,27,33^ Also, the psychometric quality of different versions of the ORTO, especially of the first ORTO-15 version, were characterized by low internal consistency.^72,73^* *In sum, because studies use different scales to measure orthorexic tendencies and the suboptimal psychometric qualities of some of these scales, the possibility to compare the outcomes across studies is limited. The development and validation of new scales to measure orthorexic tendencies could ensure higher reliability of assessment.”*

3. The review is heavily based on Western populations, which reduces cultural diversity and generalizability. This limitation should be highlighted more explicitly in the conclusions.

**We have now added this limitation to the “Strengths and Limitations” and “Conclusion” sections. It was also mentioned in the conclusion, respectively (see page 23 lines 498 to 503; page 25, lines 539-540):**
*“**The included studies were diverse in terms of location in the world, cultural groups making up the samples of participants, and language of the published research. Yet, a large number of included studies were conducted on Westernized populations,* *which shows that more research conducted among different cultural and geographical regions is needed and can help to provide a more comprehensive and holistic picture of the relationships between ON and social media.”*

*“Studies must also be done among culturally-diverse sample.”*

4. The results section contains detailed tables; however, additional synthesis (e.g., thematic grouping within tables) would improve readability.

**Table 3 was modified to group the studies in terms of the direction of the association between Orthorexia Nervosa and Social Media. The first column now shows the direction of the relationship between Orthorexia and Social Media in the first column (ON→SM; SM→ON) (see pages 52-60).**

*“Table 3. Results of Included Studies”*

5. Although the methodology section mentions the possibility of a meta-analysis, the results are purely narrative. If meta-analysis was not feasible, the reasons should be clarified.

**A justification was added in the methods section (see page 6 line 113 page 7 lines 114-115):**

*“**Given that the screened and included studies were very heterogeneous in terms of characteristics such as study design, populations, and scales used, we determined that a meta-analysis of the data was not appropriate and restricted our synthesis of the data to a narrative review. Adding to that, our main goal was to identify gaps in the literature. For that reason, a narrative scoping review was the best mean to achieve this goal.”*

6. Additionally, I recommended articles which should be added in the paper:
Bayram, H. M., Barcın-Guzeldere, H. K., Ede-Cintesun, E., & Çelik, Z. M. (2024). Exploring the interplay between social media addiction, mindful eating, intuitive eating, orthorexia nervosa, and mental health in young adults. North African Journal of Food and Nutrition Research, 8(18), 70-79. (14)
Bayram, H. M. (2024). The role of mindful eating, and intuitive eating on the relationship with orthorexia nervosa in University Students: a cross-sectional study. Revista de Nutrição, 37, e230219. (8)
Demir, H. P., & Bayram, H. M. (2022). Orthorexia nervosa: The relationship with obsessive-compulsive symptoms and eating attitudes among individuals with and without healthcare professionals. Mediterranean Journal of Nutrition and Metabolism, 15(1), 23-33. (10)

**Great suggestions. The additional references were added appropriately (see page 4 line 65; page 4 line 67; page 5 line 81).**
