# Supplementary material for: Orthorexia nervosa and social media: A mixed-methods scoping review using a systematic methodology
Source: PLoS One. 2026 Mar 11;21(3):e0340219. doi: 10.1371/journal.pone.0340219 (PMC12978498; doi:10.1371/journal.pone.0340219)
Supplement: Attachment — Submitted filename: Response_to_Reviewers_auresp_2.docx [file pone.0340219.s005.docx]

**Thank you for taking the time to provide your valuable insight. This helps us improve on our manuscript.**

Reviewer #2: 1. Abstract
• I encourage you to divide the abstract into subsections: introduction, methods, results, and conclusion.

The abstract was divided as recommended.

• Expand the results section of the abstract and provide a more concise synthesis of the methods.

Thank you for your comment. We modified based on your recommendation.

*“Results: After the identification of studies and data extraction, authors assessed the methodological quality of included studies. The characteristics and findings from the studies were narratively synthesized, with a focus on the platform, duration, and content of social media use. A total of 31 studies were identified between 2017 and 2024, which were predominantly cross-sectional and focused on Westernized populations. The results have shown a bidirectional relationship between ON and social media, influenced by the characteristics of the platform (e.g., image-based), duration of use (e.g., longer use), content themes (e.g., diet and fitness-related), and individual-level factors (e.g., limited health literacy, young adulthood and adolescence, and body dissatisfaction).”*

Lines 26-31

2. Results
• Lines 221–224: I suggest being more quantitative rather than using expressions such as “a few” or “for the most part.”

These expressions were removed to reduce ambiguity. The results reflect the consensus on the study’s quality.

*“The qualitative and mixed-methods studies included in the current review are methodologically solid with minor limitations. Meanwhile, methodological limitations in the included quantitative studies suggest moderate quality. The quality of the included studies is adequate.”*

Lines 169-171

• Move Table 2 to the Supplementary Materials and add the overall quality rating of each study to Table 1.

Table 2 was moved to the supplementary material. A column covering “Overall quality rating” was added to Table 1. A brief description was also added to the manuscript:
*“In addition, Table 1 covers the overall quality rating of each study on a scale from 1 to 7, with 7 being the best index of quality. This rating was done based on the MMAT Version 18.(20)”*

Lines 144-146

• Line 249: Instead of saying “a number of studies,” specify exactly how many studies.

This sentence was changed as recommended:
*“Previously, four studies have shown that people who spend a lot of time on social media have higher ON symptoms irrespective of the type of platform.^(27),(29),(41),(44)^.”*

Lines 189-190

• In the “Risk of Bias Assessment” paragraph, you do not need to highlight the limitations of your study; this should be done in the Discussion section.

Thank you for this feedback. The risk of bias assessment paragraph now reflects the limitations of the reviewed studies, as opposed to the limitations of our scoping review, which can be found in the limitations section.
*“Risk of bias assessment*

*Out of the reviewed studies, as detailed in Table 3, 15 reported a high risk of bias, driven primarily by significant methodological limitations. Most studies used cross sectional designs, which limits causal inferences from social media to ON. As a result, it is unknown whether social media exposure causes ON symptoms directly, or whether people with ON are more likely to engage with certain types of social media. A second limitation might be reliance on self-report measures that can generate response biases and inaccuracies in reporting ON symptoms and social media behaviors. Yet, it is most reasonable to ask individuals to self-report ON symptoms. In addition, many of the studies used non-representative samples, and were usually not representative of men nor specific cultural groups, thereby limiting the generalizability of the results to broader populations. Furthermore, there is no validated tool to measure ON across studies or social media addiction. ORTO-11 and ORTO-15 were frequently used, but have been criticized for low internal consistency and yielding different psychometric properties among varying cultures.(23, 35) In some studies, new social media use scales were used, including self-constructed items Geise (35), (36, 57), which might require further research to assess validity and reliability.”*

Lines 226-237

Furthermore, try to provide quantitative information even if you are conducting a scoping review. Avoid terms like “the majority of” or “most of” and use precise numbers instead.

Excellent observation. Numbers of studies were specified throughout the manuscript for accuracy:

*-“A total of 22 studies were quantitative, cross-sectional designs to determine the prevalence of ON symptoms and their associations with social media use.”*

Lines 159-160

-*“Out of the reviewed studies, as detailed in Table 3, 15 reported a high risk of bias, driven primarily by significant methodological limitations.”*

Lines 227-228

-*“It is also important to note that 21 of the included studies involved samples of adults only, meaning individuals over the age of 18, with only one study focusing on the relationship between ON and social media exclusively among adolescents aged between 13 and 18.(53)”*

Lines 347-349

*-“First, 19 the included studies adopted a convenience sampling method without conducting a sample size calculation, constricting the generalizability of the results within the specified population.”*

Lines 403-404

Given that you assessed 22 quantitative cross-sectional studies, this is a sufficient number to attempt a meta-analytical synthesis of the evidence. By performing a meta-analysis, you could also stratify the findings according to the type of questionnaire used for ON assessment, which would help address the limitation related to inconsistencies between ORTO-11 and ORTO-15.

We conducted a scoping review about the relationship between Orthorexia Nervosa and social media to evaluate literature gaps and inform future research. Scoping reviews are a type of knowledge synthesis, that follow a systematic approach to map evidence on a topic to identify main concepts, theories, sources, and knowledge gaps. Hence, they differ from systematic reviews and meta-analysis that primordially aim to answer a focused research question. The main aim of our study was to explore the literature relevant to Orthorexia Nervosa and social media by reviewing different definitions, and theories about the relationship found in the literature, as well as pinpoint research gaps. Scoping reviews are exploratory and descriptive in nature, which differs from systematic reviews with meta-analysis. The purpose of our scoping review and the type of data that emerged in answer to our review question are not the type of evidence that lends itself to a meta-analysis, and little value would be gained in performing such an analysis.

Given the difference in objectives and methodological approach (such as presence vs. absence of meta-analysis), scoping reviews should have different essential reporting items from systematic reviews. In our scoping review we adhered to the PRISMA extension for scoping reviews (PRISMA-ScR) where explicitly Item 13: Summary Measures is **Not Applicable**. This item from the original PRISMA is not applicable for scoping reviews because a meta-analysis is not done (that is, summary measures are not relevant). The same recommendation is echoed in JBI’s Updated methodological guidance for the conduct of scoping reviews.

Relevant references can be found below:

1. Tricco, A. C., Lillie, E., Zarin, W., O'Brien, K. K., Colquhoun, H., Levac, D., ... & Straus, S. E. (2018). PRISMA extension for scoping reviews (PRISMA-ScR): checklist and explanation. *Annals of internal medicine*, *169*(7), 467-473.

2. Rethlefsen, M. L., Kirtley, S., Waffenschmidt, S., Ayala, A. P., Moher, D., Page, M. J., & Koffel, J. B. (2021). PRISMA-S: an extension to the PRISMA statement for reporting literature searches in systematic reviews. Systematic reviews, 10(1), 39.

3. Peters, M. D., Marnie, C., Tricco, A. C., Pollock, D., Munn, Z., Alexander, L., ... & Khalil, H. (2020). Updated methodological guidance for the conduct of scoping reviews. JBI evidence synthesis, 18(10), 2119-2126.

4. Hadie, S. N. H. (2024). ABC of a scoping review: a simplified JBI scoping review guideline. Education in Medicine Journal, 16(2).
